# Rearrangement analysis of multiple bacterial genomes

**DOI:** 10.1186/s12859-019-3293-4

**Published:** 2019-12-27

**Authors:** Mehwish Noureen, Ipputa Tada, Takeshi Kawashima, Masanori Arita

**Affiliations:** 1 National Institute of Genetics, Mishima, Shizuoka 411-8540 Japan; 2 Department of Genetics, The Graduate University for Advanced Studies, SOKENDAI, Mishima, Shizuoka 411-8540 Japan; 3 RIKEN Center for Sustainable Resource Science, Yokohama, 230-0045 Japan

**Keywords:** Genome rearrangements, Reversals, *Helicobacter pylori*, Gene order

## Abstract

**Background:**

Genomes are subjected to rearrangements that change the orientation and ordering of genes during evolution. The most common rearrangements that occur in uni-chromosomal genomes are inversions (or reversals) to adapt to the changing environment. Since genome rearrangements are rarer than point mutations, gene order with sequence data can facilitate more robust phylogenetic reconstruction. *Helicobacter pylori* is a good model because of its unique evolution in niche environment.

**Results:**

We have developed a method to identify genome rearrangements by comparing almost-conserved genes among closely related strains. Orthologous gene clusters, rather than the gene sequences, are used to align the gene order so that comparison of large number of genomes becomes easier. Comparison of 72 *Helicobacter pylori* strains revealed shared as well as strain-specific reversals, some of which were found in different geographical locations.

**Conclusion:**

Degree of genome rearrangements increases with time. Therefore, gene orders can be used to study the evolutionary relationship among species and strains. Multiple genome comparison helps to identify the strain-specific as well as shared reversals. Identification of the time course of rearrangements can provide insights into evolutionary events.

## Background

Each species has a specific genome structure that changes slowly with time. In DNA sequences, local and global mutations occur. Local mutations include substitution (point mutation), insertion and deletion of a single nucleotide [1]. Global mutations (genome rearrangements) include inversion (also known as reversal), translocation, duplication, and transposition [2]. Increasing number of prokaryotic genomes and their comparison have revealed the presence of large number of genomic differences [3, 4]. Among many genomic variations, rearrangements are the most difficult to identify [5]. They affect the large segment of DNA and can occur as a consequence of different biological mechanism like DNA repair, recombination and replication [6].

Genome rearrangements change the ordering of genes. Two genomes might appear functionally identical on the basis of the gene content but their gene order can be quite different because of rearrangements. Identification of the time course of rearrangements can provide insights into evolution. The most common rearrangements that occur in uni-chromosomal genomes are inversions [1]. Genome rearrangements are rarer than point mutations and can provide useful information on the evolutionary history [7, 8].

*Helicobacter pylori* (*H. pylori*) is a Gram-negative bacterium in the human stomach and duodenum, and is used as a model organism to study human migration from Africa ~ 60,000 years ago [9–11]. Human being has been infected with *H. pylori* since its origin and more than half of the world’s population is affected [12], with different prevalence rate depending on geographical regions [13]. Genetic variability is one of the characteristics of this bacterium, to adapt and survive in different human populations [14–16]. It has an open pan genome [17], and comparison of the two strains J99 and 26695 showed that they share around 1400 core genes with rearrangements [18], and that 6 to 7% of their genes are strain specific with gene gains and losses [19–21]. Its genetic diversity is related with the history of human migration [22].

Dobzhansky and Sturtevant pioneered the genome rearrangements about 60 years ago [23], when they published an article describing the rearrangement events of 17 reversals for the species *Drosophila pseudoobscura* and *D. miranda*. Genome rearrangement is the common mode of evolution among plants, virus, mammals and bacteria [24], and Palmer et al. found that mitochondrial genomes of cabbage and turnip are very similar after reordering genes [25].

In order to identify the genome rearrangements, several algorithms have been proposed. Genome rearrangements can be represented as a series of reversals that transforms one genome into another [26]. The most widely used concept in this regard is the sorting by reversal, where genomes are represented as permutations of numbers representing distinct genes. Reversal distance for two number series is defined as the minimum number of reversals required to transform one permutation into another [27].

A computational approach for the gene order comparison was pioneered by Kececioglu and Sankoff [28]. Multiple genome rearrangement problem was considered using breakpoint distance by Sankoff and Blanchette [29], whose objective is to find the most suitable tree which best represents the rearrangement scenario [1]. Bourque and Pevzner [27] presented a greedy heuristic to create phylogenetic tree to find a reversal median.

Most of the above approaches use pairwise comparison, which transforms one genome into another assuming one as a reference and performing permutations on the other [24]. In terms of evolution, however, both genomes might have been affected by rearrangements in parallel. Therefore, multiple genome comparison is needed to identify which rearrangements are more ancestral [30]. Moreover, one of the limitations of the previous methods is that they consider fully conserved genes only.

Here, we introduce a method for identifying genome rearrangements while comparing multiple genomes of closely related strains. Our approach considers highly conserved genes present in different genomes. The method uses the orthologous gene cluster information to generate the gene order for each genome. We have used *H. pylori* strains to demonstrate the use of our method*,* as the species shows a diverse genomic structure and is a good model to study human migration across continents. Our method detects the genome rearrangements that are not only geographical region specific but also shared across continents. This analysis sheds light on not only the history of *H. pylori* but also of human beings after out-of-Africa.

## Methods

### Genome sequences

Genome sequences of 73 *H. pylori* strains were obtained from NCBI/ENA/DDBJ repository. The strains belong to 8 different geographical locations: 1) East Asia annotated as: NY40, F30, ML3, ML1, UM299, UM298, UM032, UM037, UM066, F32, oki128, XZ274, OK310, 52, F16, oki673, oki154, oki828, oki898, oki112, oki102, oki422, F57, 26695-1CH, 26695-1CL, 26695–1, Hp238, OK113; 2) South America annotated as: Sat464, Shi112, Shi169, Shi417, Cuz20, PeCan18, PeCan4, Puno120, Puno135, SJM180, v225d; 3) North America annotated as: 7C, 29CaP, Aklavik117, Aklavik86, 26695–1, 26695-1MET, J166, J99, ELS37; 4) Europe annotated as: B38, B8, HUP-B14, Rif1, Rif2, 26695, P12, 26695, G27, Lithuania75, 2017, 2018, 908; 5) Africa annotated as: SouthAfrica20, SouthAfrica7, Gambia94/24; 6) India annotated as: India7, Santal49; 7) Australia annotated as: BM013A, BM013B, BM012A, BM012B, BM012S; and 8) others of unknown location annotated as 83 and 35A. Detailed information regarding the strains is available (Additional file 1 and Table S1). One strain was registered twice: strain 26695 by TIGR and strain 26695–1 by Oita University.

### Orthologous gene clustering

Protein BLAST (version 2.2.29+, e-value<1e-5) was applied for 73 *H. pylori* strains and results were used to obtain the orthologous gene clusters through the bidirectional best-hits criterion as in our previous study [31]. For each gene cluster, its genomic position was recorded and represented as a gene table (Additional file 2: Figure S1).

### Phylogenetic analysis using core genes

Phylogenetic analysis was performed using 900 core genes of 73 *H. pylori* strains obtained from the clustering result. Core genes were aligned using MAFFT (version 7.313) [32], alignments were trimmed using trimAl [33] with default parameters, which were later concatenated and phylogenetic tree was obtained using standard-RAxML-master with the parameters: -T 11, −N 1000, −m PROTCATBLOSUM62 [34].

### Gene order identification

As reported in our previous work, strain Aklavik86 was very different from other *H. pylori* strains, maybe because of sequencing anomalies [31]. This strain was excluded from the genome rearrangement analysis. For the remaining 72 strains, gene orders were identified using the gene clusters information. The table generated with the genomic positions for each gene cluster was used as an input. Out of all the gene clusters, ‘*almost conserved*’ clusters were considered. Here, almost-conserved indicates clusters that were present in all the strains except one (see Additional file 3: Figure S2).

First, all genes in the P12 strain (used as an initial reference because analysis by Furuta et al. [35] reported no inversions in the P12 strain) were numbered from 1 to *n* in the order of their genomic positions where *n* represents the total number of genes. Genes absent in the reference strain obtained serial numbers larger than *n*. The gene order of the P12 strain was then used to obtain gene orders in other strains. In order to place ‘1’ at the start and ‘n’ at the end of all the strains, gene orders of some strains were rotated and flipped (see Additional file 4: Figure S3). After rotation and flipping, gene 1 was located at the start and gene *n* (last gene) at the end. The software program written in Python and Java is available from the author’s GitHub repository [36].

### Rearrangement identification

**Fig. 1:**
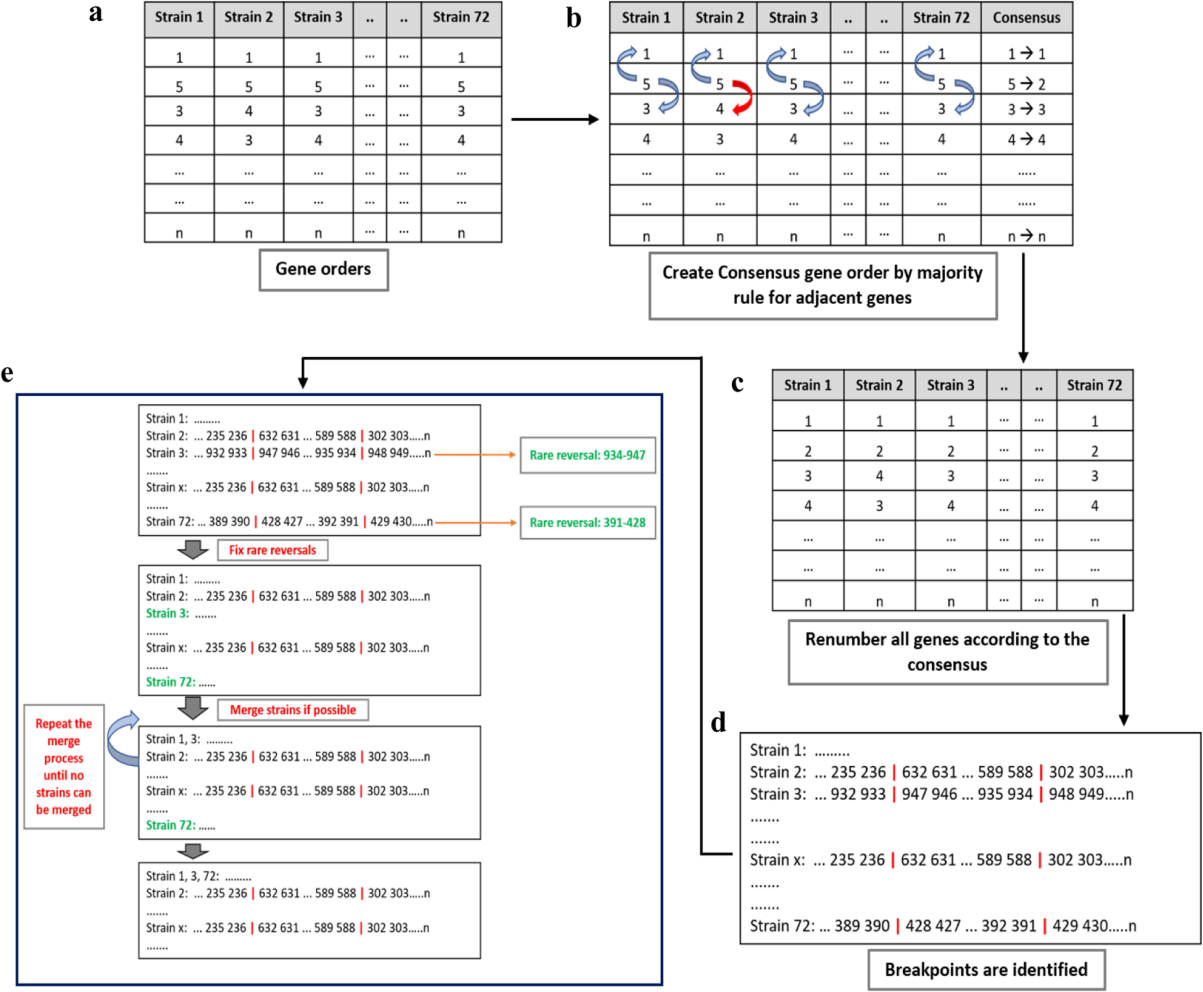
Workflow of the program. **a** Gene orders from 1 to *n*. **b** Consensus gene order created by identifying the most common upstream and downstream gene using the majority rule. Arrows (blue: majority, red: rare) indicate the upstream and downstream genes. **c** All genes reordered according to the consensus. **d** Breakpoints (red vertical lines) identified in gene orders of all strains. **e** First, the rare reversals are identified and fixed. Then similar strains are merged. Merging is repeated until no strains can be merged. Shared reversals are obtained

Rearrangements were identified as follows. 1) Creation of the consensus ordering: for each gene: the most common upstream and downstream gene are identified and the consensus gene ordering for all almost-conserved genes was created by the majority rule. Renumber all genes according to this consensus ordering. 2) Identification of breakpoints: in each strain, locations where gene numbers are gapped more than 2 are identified. Gain or loss of a single gene is not considered a breakpoint in this study. 3) Detection of rare reversals: find reversals that are observed only in a single strain and fix them. When multiple strains share the same gene ordering as a result of this process, then merge them. 4) Iteration of the merger: repeat the Step 3 (fixing and merging process) until all remaining reversals are shared. Figure 1 represents the workflow of this procedure. Remaining complex rearrangements were not resolved, and they were curated manually considering the rearrangement size and geographical regions (Additional file 5: Figure S4): inversions were basically fixed from small to larger ones for each geographical region. Inversions common in the world were postponed to resolve, because such inversions were considered closer to the tree root [36].

### Rearrangement based phylogeny

The inversions identified by the program were manually curated to obtain the phylogenetic tree reflecting the inversion history of *H. pylori*. Rearrangement based phylogeny was manually created by referencing the program output of the rearrangement identification.

## Results

### Orthologous clusters and gene orders

**Fig. 2:**
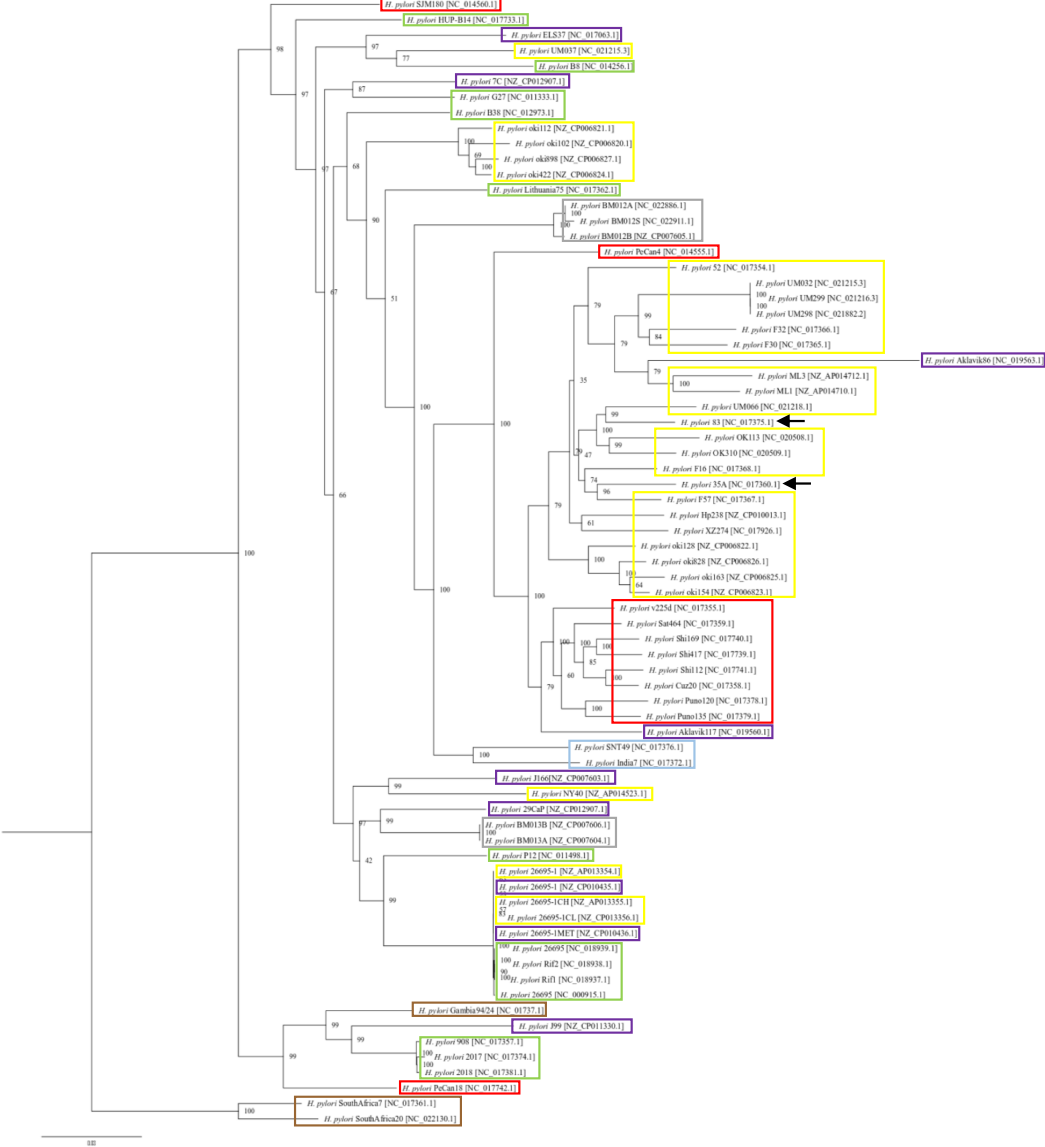
Phylogenetic tree based on the core genes of 73 *H. pylori* strains. Colored boxes represent the geographical region of the strains (Yellow: East Asia, Red: South America, Purple: North America, Green: Europe, Brown: Africa, Light Blue: India, Grey: Australia). Black arrows indicate strains with no geographical information

For 72 *H. pylori* strains (excluding Aklavik86 strain, see Methods), 1856 orthologous gene clusters were obtained. Among these 749 clusters were fully conserved (core genes) and 972 were almost-conserved gene clusters (see Methods; Fig. 2). Taking the P12 strain as a reference, gene order data for the almost-conserved gene clusters of 72 strains were identified. In this gene ordering, 15 strains did not possess the gene 1 at the start and gene *n* at the end. Among these 15 strains, gene order of 12 strains were rotated and flipped whereas gene order of 3 strains required flipping to align their gene orders (Additional file 6: Table S2). Information of strains whose gene order were rotated and flipped is given in Table 1.

**Table 1:** Information of the operation on gene order of the 15 strains

Strain	Operation on gene order	Geographical region
B8	Rotation and flipping	Europe
35A	Flipping	Not known
UM032	Rotation and flipping	East Asia
UM299	Rotation and flipping	East Asia
UM037	Flipping	East Asia
UM066	Rotation and flipping	East Asia
UM298	Rotation and flipping	East Asia
NY40	Flipping	East Asia
ML1	Rotation and flipping	East Asia
ML3	Rotation and flipping	East Asia
oki128	Rotation and flipping	East Asia
oki154	Rotation and flipping	East Asia
oki673	Rotation and flipping	East Asia
oki828	Rotation and flipping	East Asia
J99	Rotation and flipping	North America

### Rearrangement analysis

Gene order data of 72 *H. pylori* strains was used as the input (Additional file 7: Table S3). Identification of the consensus gene order was important in finding the average ordering. Renumbering of all genes using the consensus ordering revealed the positional differences of orthologous genes, which correspond to the rearrangement events. The number of breakpoints in each strain ranged from 0 to 10 (Table 2). Total 41 inversions were identified, which included strain specific as well as shared inversions. Number of inversions in each strain ranged from 0 to 6. We assumed that the strains with no inversion are closest to the tree root (not necessarily ancestral) and that the strains with 6 inversions are the farthest from the root (Table 3).

**Table 2:** Number of breakpoints identified in each strain

No. of Strains	Breakpoints	Strains annotation
6	0	P12, Shi417, Shi169, Puno135, Cuz20, Lithuania75
1	1	Aklavik117
9	2	G27, PeCan4, SJM180, Sat464, Santal49, Puno120, Shi112, BM013A, BM013B
4	3	v225d, oki154, oki673, oki828
10	4	B38, 908, F30, 2017, OK113, NY40, ML3, J99, 7C, 29CaP
8	5	B8, Gambia94/24, 2018, oki102, oki112, oki128, oki422, oki898
9	6	ELS37, 52, F57, HUP-B14, PeCan18, SouthAfrica20, ML1, J166, Hp238
2	7	SouthAfrica7, India7
17	8	26695, 35A, F16, 83, XZ274, Rif1, Rif2, 26695, OK310, UM032, UM299, UM298, 26695–1, 26695-1CH, 26695-1CL, 26695–1, 26695-1MET
0	9	–
6	10	F32, UM037, UM066, BM012A, BM012S, BM012B

**Table 3:** Number of reversals (inversions) identified in each strain

No. of Strains	No. of Reversals	Strains
7	0	Lithuania75, P12, Aklavik117^a^, Shi417, Shi169, Puno135, Cuz20
12	1	BM013A, BM013B, G27, oki154^a^, oki673^a^, oki828^a^, PeCan4, Shi112, SNT49, Puno120, Sat464, SJM180
15	2	29CaP, B38, ML3, oki128^a^, OK113, F30, v225d, 908, Gambia94/24^a^, 2017, 2018^a^, SouthAfrica20^c^, NY40, J99, 7C
13	3	B8, 52, Hp238, ML1, oki102, oki112, oki422, oki898, F57, ELS37, SouthAfrica7^b^, HUP-B14, PeCan18
9	4	OK310, UM032, UM299, UM298, XZ274, 83, 35A, F16, India7
12	5	26695, 26695–1, 26695-1MET, 26695–1, 26695-1CH, 26695-1CL, Rif1, Rif2, 26695, J166, UM066, F32
4	6	BM012A, BM012S, BM012B, UM037

^a^ignoring single gene transposition

^b^ignoring single gene transposition, 2 gene inverse transposition

^c^ignoring single gene transposition, 2 gene inverse transposition and 3 gene deletions

**Fig. 3:**
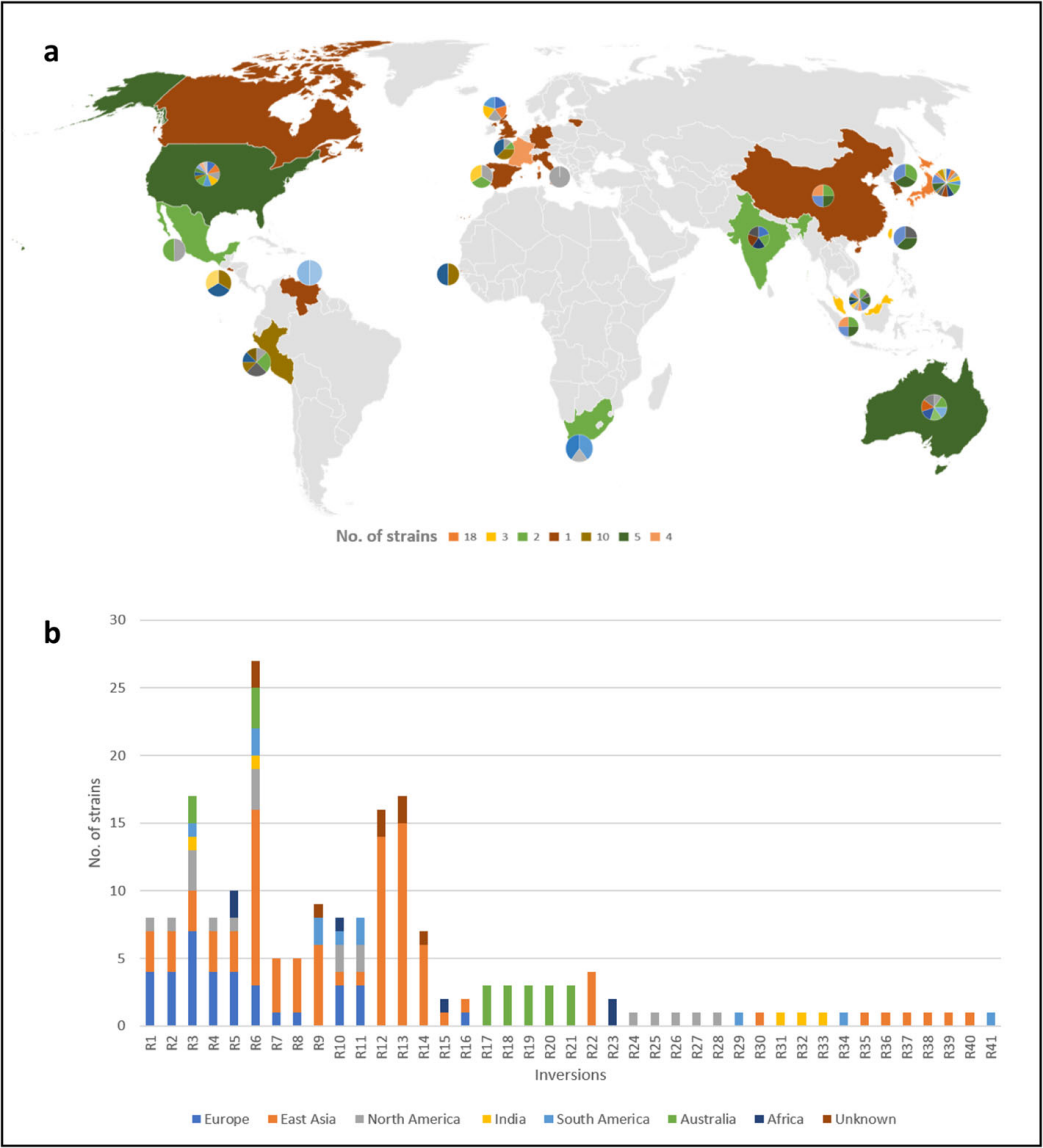
Distribution of inversions. **a** Different color of regions in the map correspond to the number of strains included in this analysis. Pie chart along each region shows the distribution of the inversions in strains of that region (Additional file 12: Table S7). **b** R1-R23 and R24-R41 were identified as shared and strain-specific inversions, respectively. Among the shared inversions, R17-R23 were region-specific

Of total 41 inversions, 18 were found strain specific whereas 23 were shared (Additional file 8: Table S4). Among all the inversions R17-R21, R22 and R23 were observed in strains from the same geographical locations such as Australia, East Asia and Africa, respectively. These inversions are called *region-specific* in this analysis. Figure 3a, illustrates the distribution of the inversions in each geographical location and Fig. 3b describes the shared, strain-specific and region-specific inversion.

Strains from Europe and East Asia shared as many as 11 inversions (R1-R8, R10, R11 and R16). Out of these 11 inversions, R7, R8 and R16 were found within them only and R1, R2 and R4 were in common with the strains from North America. Inversions R3, R5, R6, R9-R11 were shared with strains from other geographical areas (Fig. 3b). The identified inversions were of different sizes. The three large inversions (R22, R13, R8) were identified in the East Asian strains. The largest inversion (R22) was found in 4 East Asian strains from Okinawa Japan.

Furuta et al. identified inversions in 10 *H. pylori* strains and proposed a mechanism of DNA duplication linked to the chromosomal inversions [35]. Our analysis also included seven of these strains (26695, G27, P12, F16, F30, F32 and F57), and 10 inversions (R1-R4, R6, R9, R12-R14 and R30) identified in these strains were similar to those reported by Furuta et al. (Additional file 9: Table S5) [35]. Since we did not perform analysis at the DNA sequence resolution, true identity of inversion requires further sequence-level analysis. For 29 strains, inversion breakpoints were examined to identify the possible cause of the rearrangements. 19 strains possessed insertion sequences (IS), 10 possessed integrated conjugative elements (ICEs), and 7 possessed virulence related genes and pathogenicity island proteins around their inversions breakpoints (Additional file 10: Table S6).

### Rearrangement hotspots

Some regions were frequently involved in rearrangements and called *‘rearrangement hotspots’* [9, 17]. Three such regions were identified in the analyzed strains. Breakpoints within these regions were found to have IS, ICE, repeats, virulence related genes and restriction modification system proteins. Even if two inversions share a common breakpoint, however, the mobile elements around them were sometimes different or strain-specific (Additional file 11: Figure S5).

### Phylogenetic tree based on inversions

**Fig. 4:**
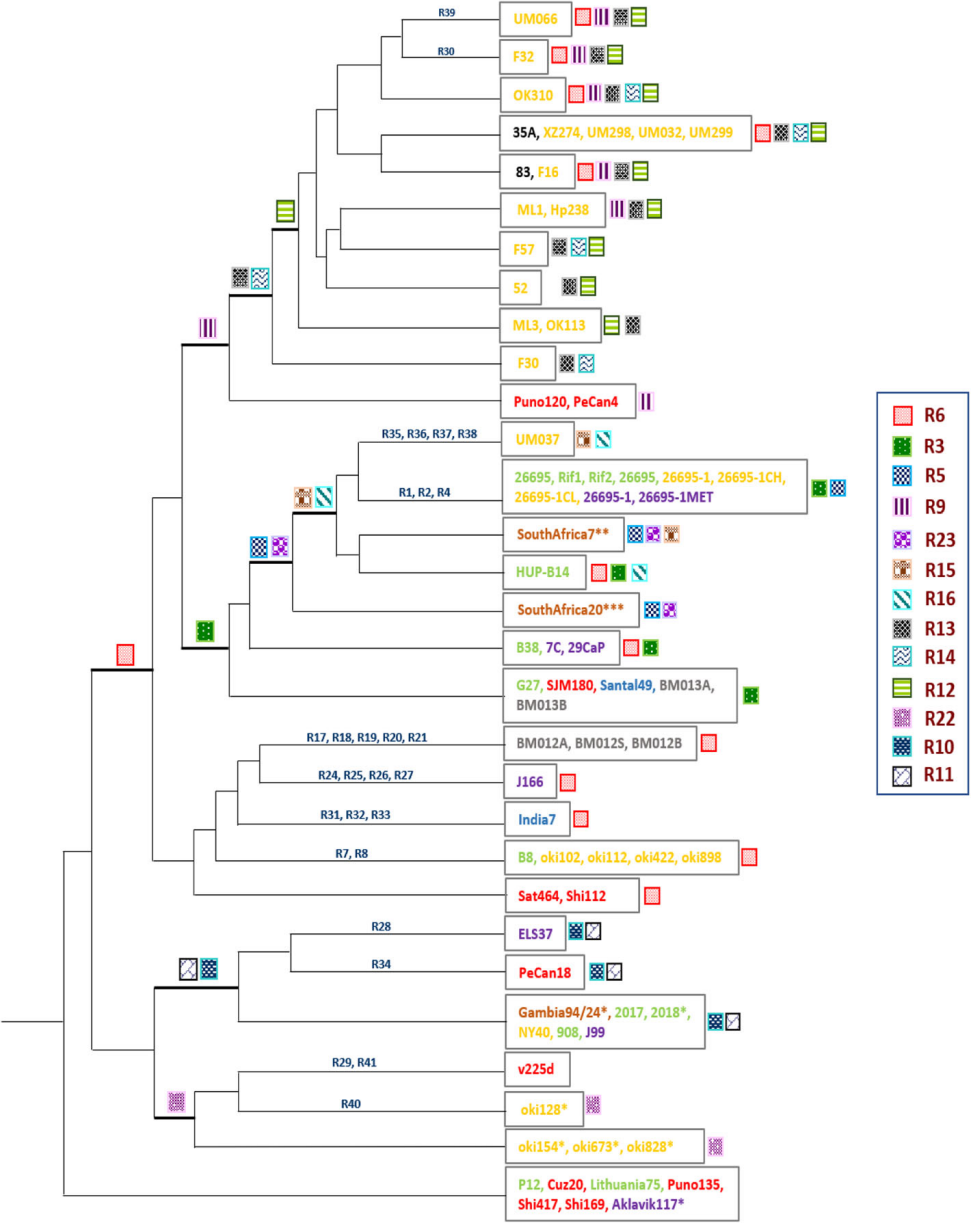
Inversion-based phylogeny. Labels beside the branches represent the inversions occurred in the strains (Additional file 8: Table S4). Strains names are colored representing the geographical location (same as Fig. 2). Strains name in black color show the strains with no geographical information. Legend on the right side indicate the reversals shared among multiple strains. * ignoring single gene transposition, ** ignoring single gene transposition and 2 gene inverse transposition, *** ignoring single gene transposition, 2 gene inverse transposition and 3 gene deletion

Information of inversions that occur during the evolution was used to create a phylogenetic tree. First, the matrix representing the presence or absence of all inversions in each strain was constructed (Additional file 12: Table S7). Then the tree was created to reflect the evolution of *H. pylori* strains from different geographical locations (Fig. 4).

Some of the inversions (R3, R6, R9, R12, R13 and R14) occurred more frequently and were present in multiple strains. R3 and R6 were found in strains from all geographical locations except for Africa. R9 was found in strains from South America, East Asia and Africa. R12, R13, R14 occurred in strains from East Asia and in strains with no geographical information. R10 and R11 occurred less frequently and were present in strains from all geographical locations except for India.

Inversions can be classified into two types: shared and specific inversions. The frequent inversions are regarded as shared, and the less frequent, specific. Strains from East Asia mostly showed the shared rearrangements whereas few strains had both types. Strains from South America and Europe mostly showed the shared rearrangements with few exceptions: PeCan18 strain (from South America) and B8, 26695, Rif1, Rif2, 26695 strains (from Europa) had both shared and specific rearrangements; v225d strain from South America had only specific rearrangements. Three strains from North America had the shared rearrangements only whereas four strains had both types. One strain (UM037) from East Asia and three strains (BM012A, BM012B, BM012S) from Australia had greater number of strain specific inversions compared to other strains. These strains were the most rearranged with six inversions: two or one shared and four or five specific inversions, respectively.

For some of the 72 *H. pylori* strains, information regarding the disease states of isolated patients was available: 8 from duodenal ulcer, 4 from gastritis, 4 from MALT lymphoma, 5 from gastric atrophy, 4 from peptic ulcer and 8 from gastric cancer (Additional file 1: Table S1). East Asian group included strains isolated from the patients having almost all of the mentioned disease states, from duodenal ulcer to gastric cancer. Of the eight strains isolated from cancer patients, two strains (PeCan18, ELS37) had two (R10, R11) shared and one (PeCan18: R34, ELS37: R28) specific inversion, whereas four other strains (2017, 2018, 908, J99) isolated from duodenal ulcer patients possessed the similar shared inversions (R10, R11) but no specific inversion. From this, we can infer that the specific inversion in strains PeCan18 and ELS37 might be associated with cancer. The remaining six cancer strains (F32, XZ274, F57, PeCan4, 7C, 29CaP) shared inversions except for F32 which had only one specific inversion (R30). The list of shared inversions in each strain were: F32: [R6, R9, R12, R13], XZ274: [R6, R12, R13, R14], F57: [R12, R13, R14], PeCan4: [R9], 7C: [R3, R6] and 29CaP: [R3, R6]. Although several strains shared same inversions, these inversions may be historically independent. More detailed sequence-level analysis is necessary to confirm the identity of inversions.

## Discussion

Degree of genome rearrangements increases with time as point mutations accumulate, both reflecting the evolutionary history of genomes. The number of inversions in *H. pylori* genomes was far less than the number of strain-specific genes, not to say of point mutations. Inversions therefore tells evolutional history in a longer timescale.

Among the 41 identified rearrangements, many were specific and few were geographic region-related. Although the investigated number of *H. pylori* genomes was too small to grasp the human migration, many rearrangements were not shared within regions partly because insertion sequences or virulence genes induce similar inversions. This also suggests that some inversions are associated with disease states irrespective of geography (or human migration), and certain inversions were linked with gastric cancer in our analysis. The pattern of inversions was most diverse in Japan (Fig. 3a) probably because of the larger number of sampling. The North American region also had the diverse inversion pattern (Fig. 3a) even though the number of samples was much smaller compared to Japan. This diversity occurred maybe because of human migration. Since our analysis is based on orthologs and not the entire genomic region, verification needs more in-depth analysis using the whole genome sequences.

The obvious benefit of our method is scalability: whole genome comparison is difficult for many genomes using previous approaches comparing two genomes. Our method can handle hundreds of strains at the level of gene orders. In terms of methodology, our simple approach does not resolve some complex rearrangements automatically, and they were later resolved manually. We are working on the automation of resolving the complex rearrangements and visualization of phylogeny in a much larger scale.

## Conclusion

Gene orders can be used as a measure to study the evolutionary relationship of species. Previous studies considered only fully conserved genes in the pairwise comparison. Our approach considers conserved gene clusters in a large number of genomes and identifies their rearrangements. Many inversions in *H. pylori* strains were shared across geographic regions, and only few were found to be geographic region-specific. Some inversions were associated with disease states such as cancer, so analyzing *H. pylori* genomes on a larger scale more in details can help us to understand the disease mechanism. Since *H. pylori* has evolved with the global human migration, studying inversions may reveal the migration pattern although few rearrangements were geography related.

## Supplementary information

**Additional file 1:** **Table S1.** 72 *Helicobacter pylori* strains information.

**Additional file 2:** **Figure S1.** Gene clusters recorded in the form of table.

**Additional file 3:** **Figure S2.** Example of almost conserved gene clusters.

**Additional file 4:** **Figure S3.** Example of gene order rotation and flipping.

**Additional file 5:** **Figure S4.** Complex rearrangement

**Additional file 6:** **Table S2.** Gene order of 72 *Helicobacter pylori* strains before and after rotation and flipping.

**Additional file 7:** **Table S3.** Consensus based gene order of 72 *Helicobacter pylori* strains.

**Additional file 8:** **Table S4.** Information of the 41 identified inversions.

**Additional file 9:** **Table S5.** Rearrangements that were in common with previous study by Furuta et al [35].

**Additional file 10:** **Table S6.** Information of IS, ICE, virulence related genes and pathogenicity island proteins around some of the inversions breakpoints.

**Additional file 11:** **Figure S5**. Identified rearrangement hotspots.

**Additional file 12:** **Table S7.** Matrix representing the presence or absence of inversions in each strain. Legend of the pie charts in Fig. 3a.

## Data Availability

The complete genome sequences used in this study are available at GenBank (See Additional file 1: Table S1 for the accession numbers). The program codes are available at GitHub (https://github.com/mehwish-noureen/Reversals_identification).

## Abbreviations

ICE: Integrated conjugative element
IS: Insertion sequence
NCBI/ENA/DDBJ: National Center for Biotechnology Information/European Nucleotide Archive/DNA Data Bank of Japan
TIGR: The Institute for Genomic Research
