# Supplementary material for: Rearrangement analysis of multiple bacterial genomes
Source: BMC Bioinformatics. 2019 Dec 27;20(Suppl 23):631. doi: 10.1186/s12859-019-3293-4 (PMC6933940; doi:10.1186/s12859-019-3293-4)
Supplement: Supplementary file 2 — Additional file 2: Figure S1. Gene clusters recorded in the form of table. [file 12859_2019_3293_MOESM2_ESM.pdf]

Genomes

Gene clusters

	Strain 1	Strain 2	Strain 3	Strain 4	..	Strain 72
Gene A	216	0	12	104	...	50
Gene B	634	418	430	522	...	468
Gene C	NA	NA	910	1002	...	948
Gene D	1114	1715	1727	NA	...	1765
Gene E	1931	2502	2514	NA	...	NA
...	..	..	...	...	...	...
Gene XXXX	11586	11359	11470	11462	...	11699

→ Genomic positions

Figure S1. Each row represents one cluster, each column represents a strain and each cell represents the genomic position of a gene in a particular strain. NA indicates the gene is absent in particular strain.
