# Supplementary material for: Rearrangement analysis of multiple bacterial genomes
Source: BMC Bioinformatics. 2019 Dec 27;20(Suppl 23):631. doi: 10.1186/s12859-019-3293-4 (PMC6933940; doi:10.1186/s12859-019-3293-4)
Supplement: Supplementary file 3 — Additional file 3: Figure S2. Example of almost conserved gene clusters. [file 12859_2019_3293_MOESM3_ESM.pdf]

	Strain 1	Strain 2	Strain 3	Strain 4
Gene A	216	0	NA	104
Gene B	634	418	430	522
Gene C	NA	NA	910	1002
Gene D	1114	1715	1727	NA
Gene E	1931	2502	2514	2434

Figure S2. Example of almost conserved gene clusters in four strains. The clusters encircled in red are the almost conserved gene clusters as they are present in all strains except one.
