# Supplementary material for: Rearrangement analysis of multiple bacterial genomes
Source: BMC Bioinformatics. 2019 Dec 27;20(Suppl 23):631. doi: 10.1186/s12859-019-3293-4 (PMC6933940; doi:10.1186/s12859-019-3293-4)
Supplement: Supplementary file 4 — Additional file 4: Figure S3. Example of gene order rotation and flipping. [file 12859_2019_3293_MOESM4_ESM.pdf]

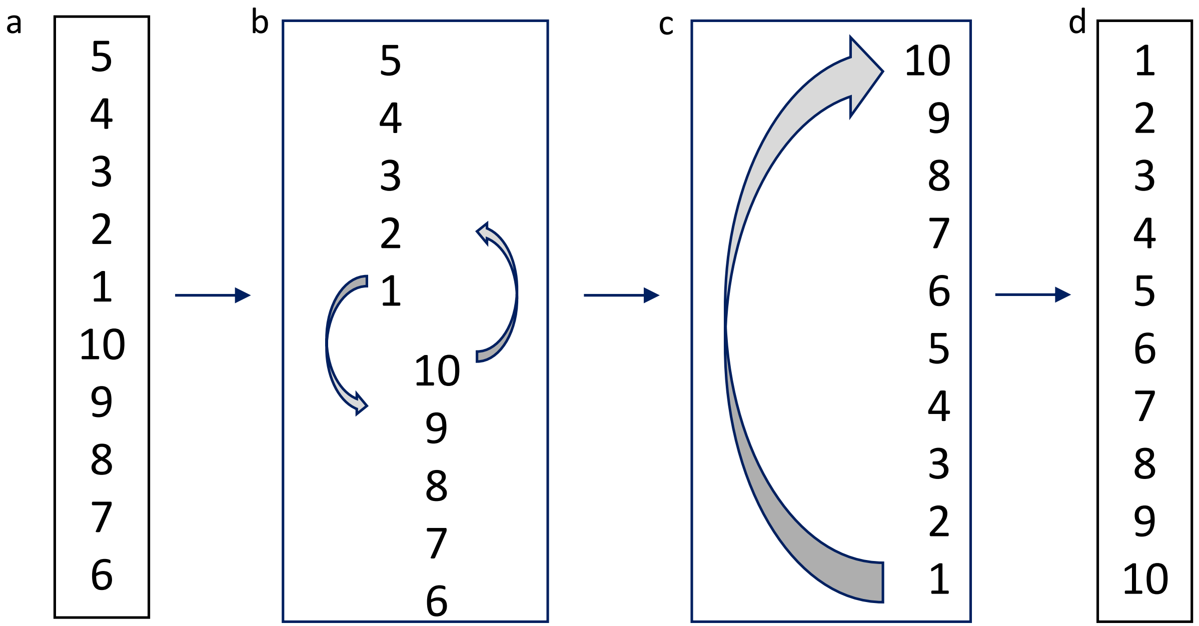

Figure S3. Gene order rotation and flipping example. **a** Gene order neither starting from 1 nor ending at 10 (maximum number here). **b** Gene order flipped. **c** Gene order rotation. **d** Gene order after flipping and rotation.
