# Supplementary material for: Rearrangement analysis of multiple bacterial genomes
Source: BMC Bioinformatics. 2019 Dec 27;20(Suppl 23):631. doi: 10.1186/s12859-019-3293-4 (PMC6933940; doi:10.1186/s12859-019-3293-4)
Supplement: Supplementary file 5 — Additional file 5: Figure S4. Complex rearrangement [file 12859_2019_3293_MOESM5_ESM.pdf]

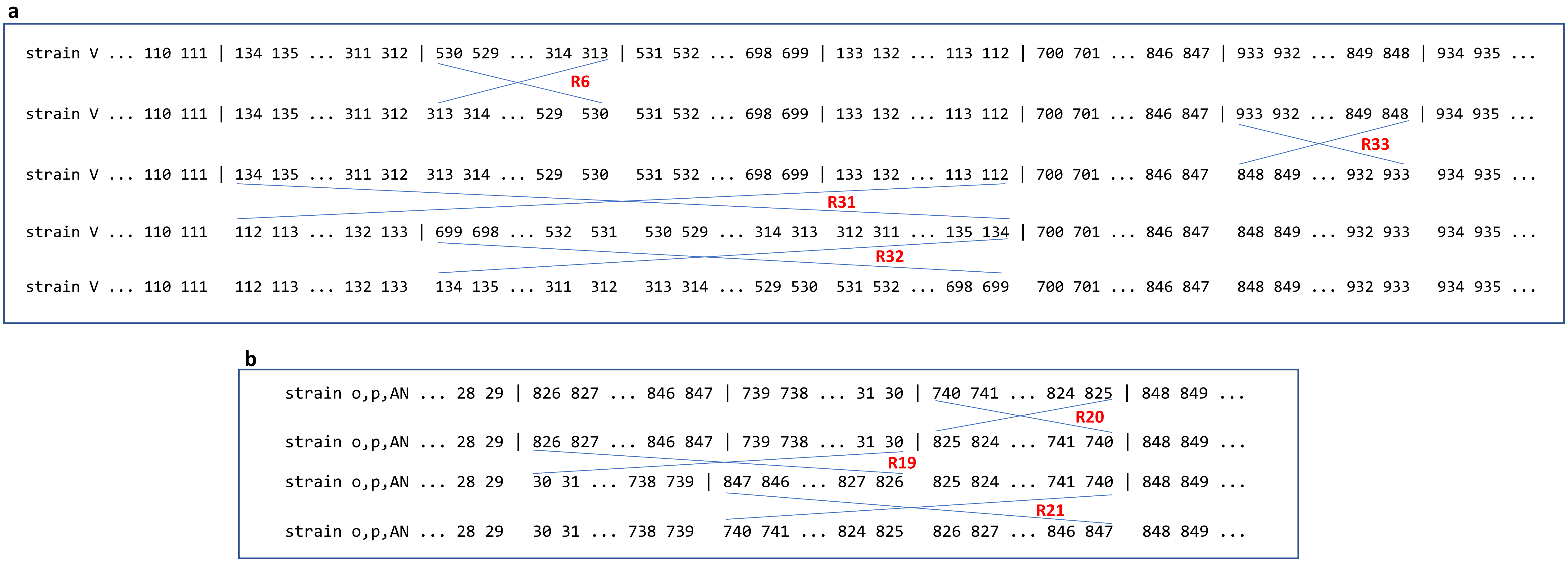

Figure S4: Complex rearrangements. **a, b)** Inverted regions are resolved in the order of number of breakpoints within it (lowest to highest)
