# Supplementary material for: Rearrangement analysis of multiple bacterial genomes
Source: BMC Bioinformatics. 2019 Dec 27;20(Suppl 23):631. doi: 10.1186/s12859-019-3293-4 (PMC6933940; doi:10.1186/s12859-019-3293-4)
Supplement: Supplementary file 11 — Additional file 11: Figure S5. Identified rearrangement hotspots. [file 12859_2019_3293_MOESM11_ESM.pdf]

a

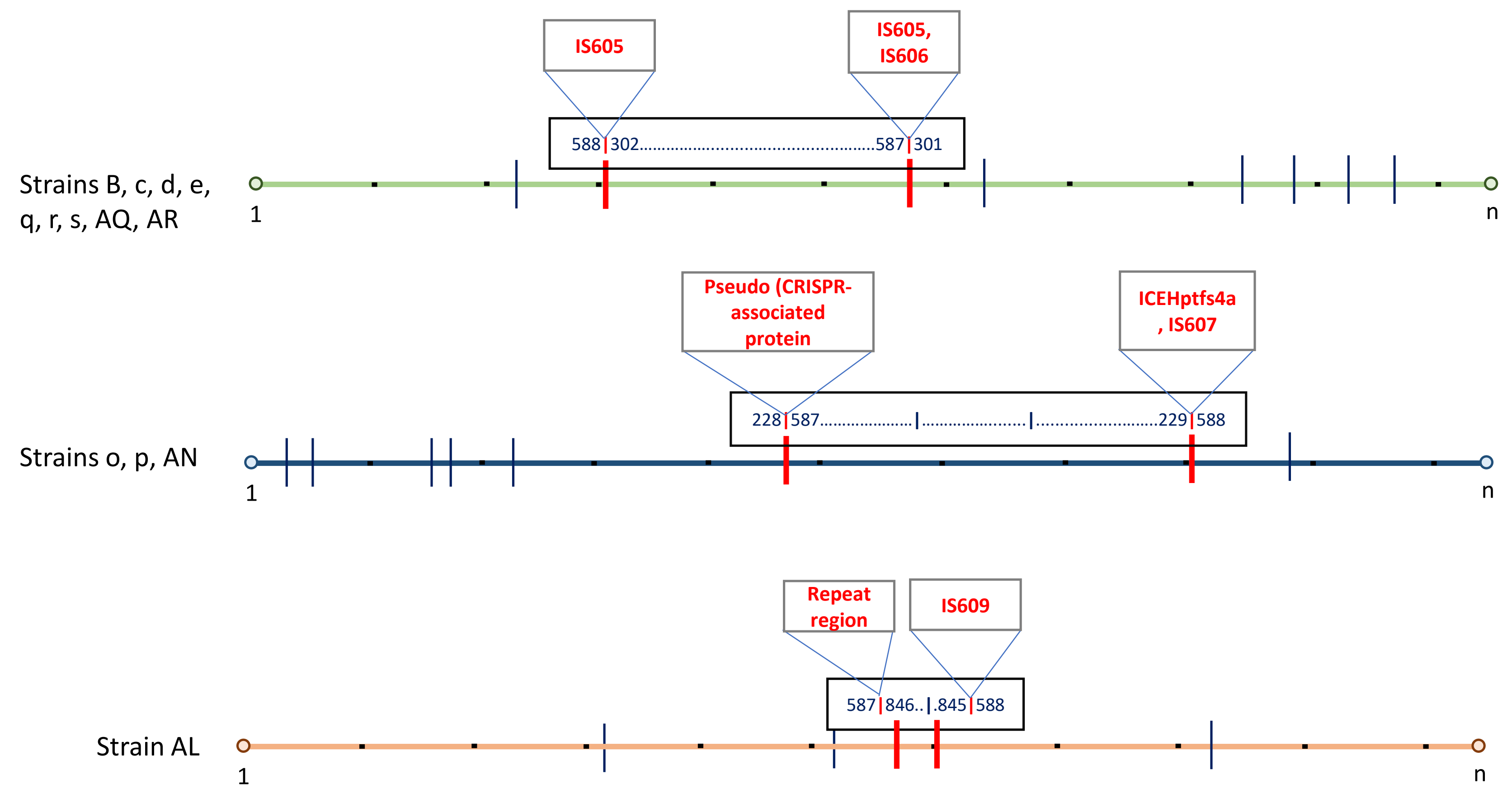
IS605
IS605,
IS606
588 302 587 301
Strains B, c, d, e,
q, r, s, AQ, AR
1
n
Pseudo (CRISPR-
associated
protein
ICEHptfs4a
, IS607
228 587 229 588
Strains o, p, AN
1
n
Repeat
region
IS609
587 846.. .845 588
Strain AL
1
n

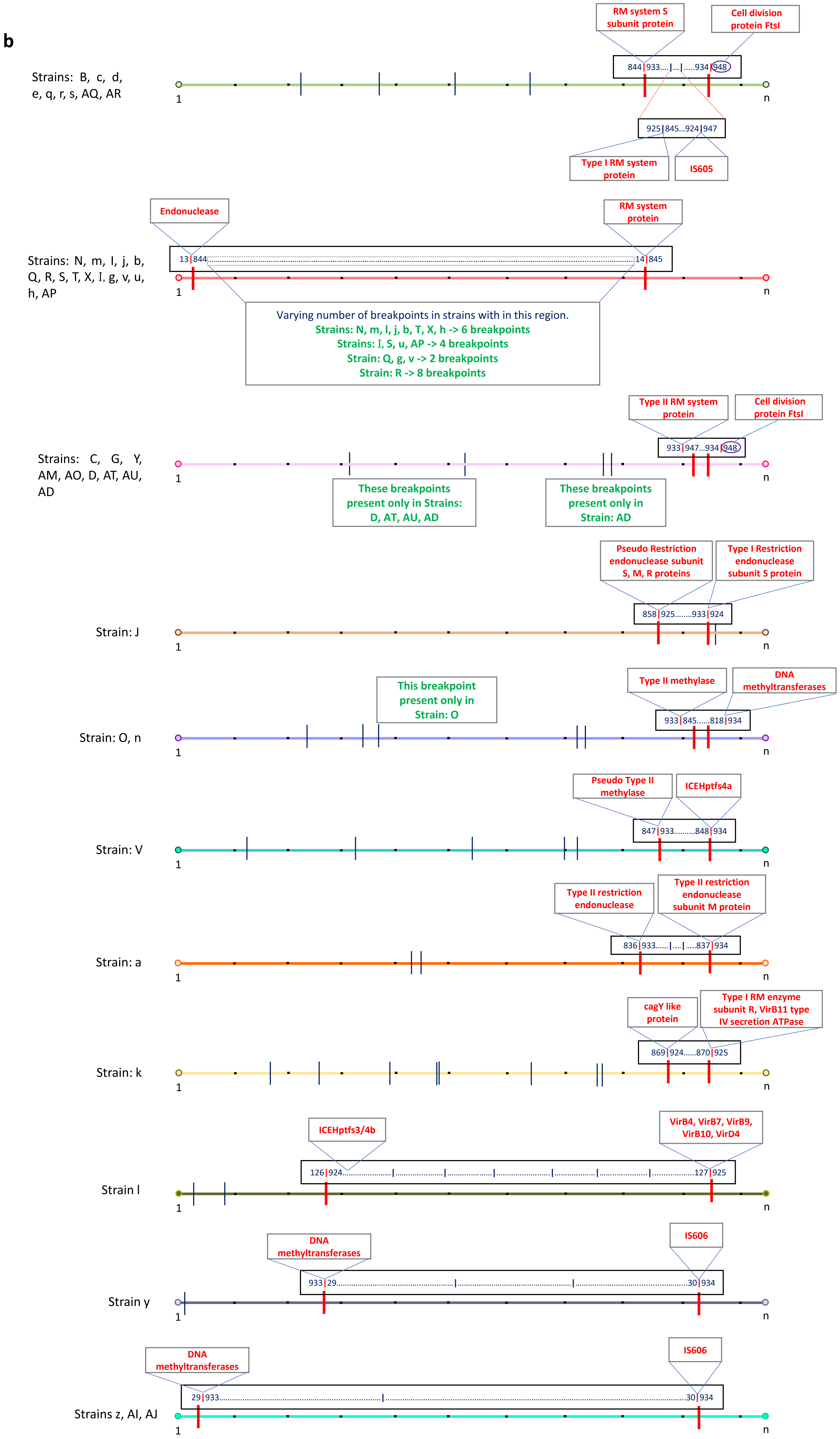

b
Strains: B, c, d, e, q, r, s, AQ, AR
RM system S subunit protein
Cell division protein FtsI
844|933....|...|.....934|948
925|845...924|947
Type I RM system protein
IS605
Strains: N, m, l, j, b, Q, R, S, T, X, I, g, v, u, h, AP
Endonuclease
RM system protein
13|844
14|845
Varying number of breakpoints in strains with in this region.
Strains: N, m, l, j, b, T, X, h -> 6 breakpoints
Strains: I, S, u, AP -> 4 breakpoints
Strain: Q, g, v -> 2 breakpoints
Strain: R -> 8 breakpoints
Strains: C, G, Y, AM, AO, D, AT, AU, AD
Type II RM system protein
Cell division protein FtsI
933|947...934|948
These breakpoints present only in Strains: D, AT, AU, AD
These breakpoints present only in Strain: AD
Strain: J
Pseudo Restriction endonuclease subunit S, M, R proteins
Type I Restriction endonuclease subunit S protein
858|925........933|924
Strain: O, n
This breakpoint present only in Strain: O
Type II methylase
DNA methyltransferases
933|845......818|934
Strain: V
Pseudo Type II methylase
ICEHptfs4a
847|933.........848|934
Strain: a
Type II restriction endonuclease
Type II restriction endonuclease subunit M protein
836|933......|.....|......837|934
Strain: k
cagY like protein
Type I RM enzyme subunit R, VirB11 type IV secretion ATPase
869|924......870|925
Strain l
ICEHptfs3/4b
VirB4, VirB7, VirB9, VirB10, VirD4
126|924
127|925
Strain y
DNA methyltransferases
IS606
933|29
30|934
Strains z, AI, AJ
DNA methyltransferases
IS606
29|933
30|934
1
n

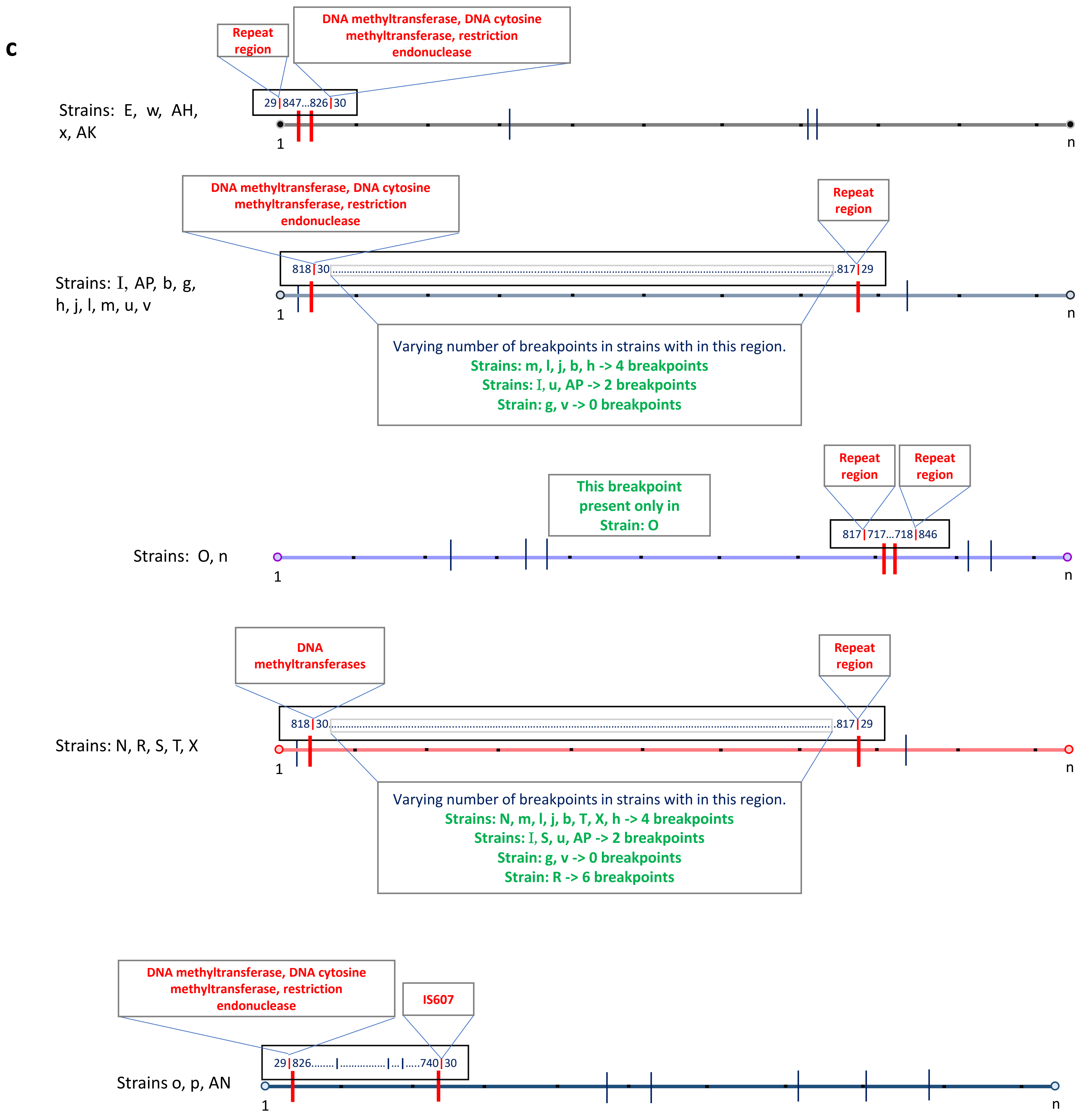

Figure S5: Rearrangement hotspot. The breakpoints [588, 587] in **a**, [(844, 845), (933, 934), (924, 925)] in **b** and [(29, 30), (818)] in **c** reflect the region involved in different rearrangements. The blue vertical lines indicate the other breakpoints present in each strain. The red vertical lines indicate the region (breakpoint) called the hotspot. The boxes show the different elements present around these breakpoints.
